# Microbeam two-dimensional small-angle X-ray scattering investigating the effects of reduced graphene oxide on local microstructures of high-density polyethylene/reduced graphene oxide nanocomposite bars

**DOI:** 10.1098/rsos.181866

**Published:** 2019-02-13

**Authors:** Guibin Yao, Tianchen Duan, Enyi Chi, Pengran Guo, Yiguo Li, Zongbao Wang

**Affiliations:** 1 Ningbo Key Laboratory of Specialty Polymers, Faculty of Materials Science and Chemical Engineering, Ningbo University, Ningbo 315211, People's Republic of China; 2 Guangdong Provincial Key Laboratory of Emergency Test for Dangerous Chemicals, China National Analytical Center, Guangzhou 510070, People's Republic of China

**Keywords:** high-density polyethylene, reduced graphene oxide, epitaxial crystallization, nanocomposite, microbeam

## Abstract

It has been reported that the introduction of reduced graphene oxide (RGO) can enhance the crystallization and orientation of high-density polyethylene (HDPE) matrix and thus improve the mechanical properties of HDPE/RGO nanocomposites. In this study, the local microstructures and orientations in different regions of HDPE/RGO bars with varied RGO contents were further explored by two-dimensional small-angle X-ray scattering using a microbeam technique. It is unveiled that the orientation orderings of each position is intensified with increasing RGO amount, and of particular interest is the observation of the slight change of the ordering degrees in diverse zones of HDPE/RGO nanocomposite bars, indicating that RGO imposes a uniform enhancing effect upon HDPE matrix within different areas and consequently induces an effective increase of the mechanical properties of HDPE/RGO nanocomposites.

## 1. Introduction

Polymeric materials have been widely used in a number of fields due to their unique characteristics. Most of the products are manufactured by polymer processing procedures such as injection moulding, fibre spinning and extrusion. Among these, injection moulding is widely employed for shaping the products and a large number of researchers have devoted themselves to studying and modelling the injection moulding process for thermoplastics [1–4]. The fact that the flow drastically affects the crystallization kinetics and crystal morphology of semicrystalline polymers during injection processing [5–9] cannot be ignored. It is commonly recognized that the shear flow enhances the crystallization process and thus affects the properties of polymers by improving the orientation of polymer chains [10–12]. For example, a unique kind of skin-core structure has been disclosed in the injection moulded isotactic polypropylene (iPP) and high-density polyethylene (HDPE) bars [13–17]. It is widely accepted that the skin-core structure can be divided into three parts: highly oriented skin layer, shear layer made up of highly oriented molecules along the flow direction, and spherulitic core without chain orientation. The skin layer and shear layer, consisting of highly orientated folded chain lamellae and extended chain crystals, are the main structures supporting the polymer's mechanical performance although the total width of skin layer and shear layer is generally not large [18]. The morphology, orientation and their distribution of macromolecules at distinct zones are different due to their complex thermomechanical history, which results in the highly anisotropic and nonhomogeneous structures, affecting the physical behaviour and ultimate mechanical properties of injection-moulded parts [19,20]. Although extensive studies have been conducted on the structures and properties of injection bars of pure polymers [13,21,22], much less work has been reported on the promising novel polymer/nanofiller composites.

It is now well known that carbon-based nanofillers, such as diamond and graphite, can particularly enhance the crystallization, orientation and mechanical properties of semicrystalline polymers [23]. As the first 2D counterpart of graphite with single sheet and as the strongest material, graphene has been widely used [24,25]. Lots of studies have shown that graphene can improve the mechanical properties of polymers [26,27]. It has already been confirmed that reduced graphene oxide (RGO) can induce the epitaxial crystallization of semicrystalline poly(ɛ-caprolactone) (PCL) chains upon the RGO surfaces [28,29]. As noted above, the shear flow during injection moulding can also enhance the crystallization and mechanical properties of polymer injection bars [10–12]. It is, therefore, highly interesting to disclose the dual impacts of shear flow and nanofillers on the structure and properties of injection bars of polymer/nanofiller composites.

Recently, we have reported the enhancing effect of introducing RGO on the mechanical properties of injection bars of HDPE/RGO nanocomposites [30]. The structural examination of the centre of injection bars has proved that the intensification can be attributed to the dual role of the flow field and epitaxial crystallization of HDPE on RGO surfaces [30]. However, to deepen the understanding of the coupling effect of the shear flow and RGO, it is important to detect the microstructures of injection bars of HDPE/RGO nanocomposites. Microbeam X-ray scattering is a powerful tool for gathering the structural information on local spatial inhomogeneity of nanostructures in the micrometre scale and thus provides an important way to achieve an in-depth understanding of the impact of polymer structures on the product properties [31–33]. For instance, scanning microbeam X-ray scattering measurements have been successfully applied to analyse the microstructure of the injection-moulded iPP [15,34–36]. In this work, using the microbeam X-ray scattering technique, the microstructures of the injection bars of HDPE/RGO nanocomposites at different regions from the edge to the core were investigated to unveil the structural evolution regularity and then to further understand the mechanical enhancing effect of RGO on the injection bars of HDPE/RGO nanocomposites.

## 2. Material and methods

### 2.1. Experimental materials

The HDPE, with an average weight *M*_n_ of 15820 g mol^−1^ and a polydispersity index *PDI* of 4.9, was purchased from Dow Chemical Company. Natural flake graphite with a mean particle size of 50 µm was purchased from Qingdao Jiuyi graphite Co., Ltd. (Shandong, China). Hydrochloric acid (HCl) (37%), sulfuric acid (H_2_SO_4_) (98%), potassium nitrate (KNO_3_), potassium permanganate (KMnO_4_), hydrogen peroxide (H_2_O_2_) (35%), *n*-hexanol and chloroform were purchased from Sinopharm Chemical Reagent Co., Ltd. (Shanghai, China). All reagents were used as received without purification.

### 2.2. Sample preparation

Graphene oxide (GO) was exfoliated by ultra-sonication from graphite oxide which was produced by modified Hummers' method [37]. RGO was prepared by the thermal exfoliation and reduction of GO [38]. The sizes of standard injection bars of HDPE/RGO nanocomposites with different RGO contents (0, 0.1, 0.5 and 1.0 wt%) were 50 mm × 4 mm × 2 mm. First, the raw materials of HDPE and RGO were added into the injection moulding machine (HAAKE MiniLab) with a cavity temperature of 180°C. Then, the mixing process was conducted under a screw speed of 80 r min^−1^ for 5 min. Finally, the blends were extruded into the mould whose temperature and pressure were preset at 80°C and 650 bar, respectively, and the moulding process lasted 1 min after the injection. Because of the location of the gate at one end of the mould, the melt flowing into the mould cavity was parallel to the length direction of the mould, i.e. the resulting injection bars.

### 2.3. Analytical methods

Two-dimensional small-angle X-ray scattering (2D SAXS) experiments were carried out on the BL16B1 beam-line in the SSRF with beam sizes of 25 m × 25 µm, and the wavelength of the monochromatic X-ray was 1.24 Å. The 2D patterns were recorded in the transmission mode at room temperature with a sample-to-detector distance of 2000 mm. The samples were translated through the microbeam stepwise, and SAXS patterns recorded at each step. The width of all samples was 4000 µm. Therefore, we scanned the sample using X-ray microbeam from a distance of 400 µm from the edge to the centre which is 2000 µm away from the edge. The step size was 400 µm along the direction perpendicular to flow field and the exposure time was 100 s.

All X-ray images were corrected for background scattering, air scattering and beam fluctuations. The SAXS data analysis was performed using the Fit2d software package [39]. The lamellae parameters derived from the SAXS data are named the long period *L*. It can be calculated according to the Bragg equation

2.1 $$L=\frac{2\pi }{q^{*}},$$

q* represents the peak position in the scattering curves.

## 3. Results and discussion

**Figure 1.**
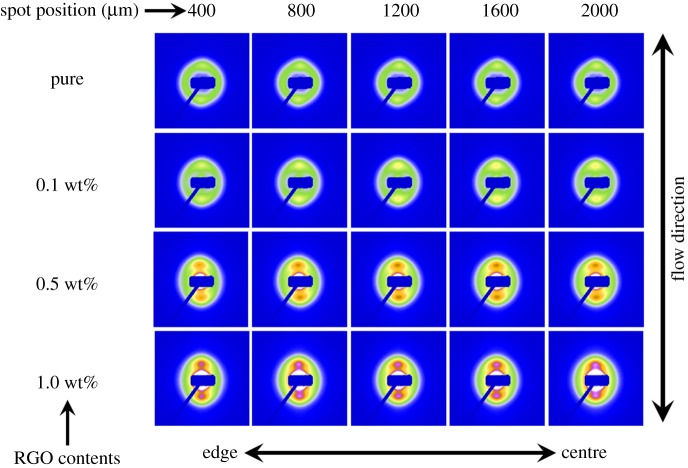
Two-dimensional microbeam SAXS patterns for HDPE/RGO nanocomposites with different RGO contents and obtained five points from the edge 400 µm distance to the centre of the sample.

It is well known that 2D SAXS patterns can reflect different polymer structures. The 2D SAXS patterns of isotropic samples typically display a strong scattering ring, while an oriented sample often shows the strong two-point patterns with the maximum in the draw or flow direction or within a certain angle about the reference direction [40]. Figure 1 gives the 2D SAXS patterns for the bars of pure HDPE and its nanocomposites with different RGO contents at different regions. It can be seen that the pure HDPE and its nanocomposites with different RGO contents in different positions all display two small discernible scattering spots, but the pure HDPE bar shows the rather poor scattering signal. To facilitate discussion, we selected the sample with 1.0 wt% RGO (i.e. the fourth line) and the positions of 800 µm to the outmost edge (i.e. the second column) as examples to discuss. According to the fourth line, it is evident that the changes in the same sample are not obviously with the increase in distance to the edge. However, for the same position, the phenomenon is quite different with increasing RGO content. It can be seen from the second column that an almost isotropic reflection circle and two small scattering spots appear on the outer ring of the pure HDPE. But for HDPE/RGO nanocomposites, the small scattering spots gradually become the bulb-shaped lobes in the flow direction with the increase of RGO content. Moreover, the scattering disks change from circular ring for pure HDPE to elliptical ring for HDPE/RGO nanocomposites. Meanwhile, it is worth pointing out that the periodic structure appears at the centre of the SAXS patterns for HDPE/RGO nanocomposites. Compared with the pure HDPE, which has no periodic structure at the centre, the periodic structure is enhanced in the equator and meridian direction with the increase of RGO content. Comparing the scattering patterns at different positions in the same sample, the change is not obvious with the increase of distance away from the edge. These results show that the orientation of the sample is dramatically enhanced with the increase of RGO content, irrespective of different regions. But the enhancement is not obvious from the edge to the centre of the same injection bar.

**Figure 2.**
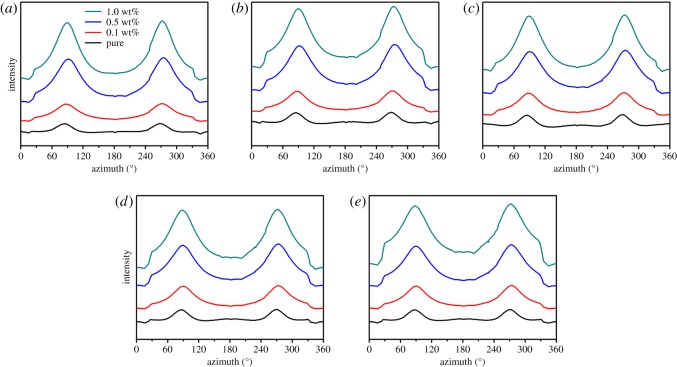
Azimuthally integrated profiles of pure HDPE and its nanocomposites with different RGO contents at the location of the different distance to the sample edge: (*a*) 400 µm, (*b*) 800 µm, (*c*) 1200 µm, (*d*) 1600 µm and (*e*) 2000 µm.

To investigate the orientation of HDPE matrix, SAXS intensity profiles of pure HDPE and its nanocomposites are further presented. Figure 2 shows azimuthally integrated profiles of all samples. It can be observed that two weak peaks at different positions of pure HDPE are parallel to the injection flow direction, which indicates that the flow field can promote the orientation of HDPE crystals. The orientation degree of HDPE chains is enhanced significantly in the meridian direction and increases dramatically with the increase of RGO contents. It is very clear that this phenomenon occurs in all different points. This suggests that the influence of RGO on the HDPE injection bars is comprehensive.

**Figure 3.**
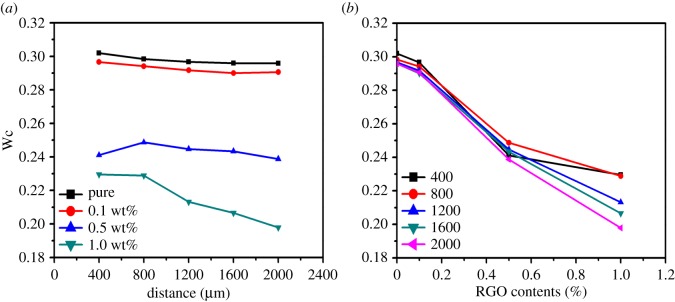
FWHM profiles (*a*) different distances to the outermost of pure HDPE and its nanocomposites with various RGO contents, (*b*) same positions of Pure HDPE and its nanocomposites with different RGO contents.

For wide angle X-ray diffraction, the degree of crystalline ordering, including the packing order and crystal size, can be estimated from the full width at half maximum (FWHM) of a diffraction peak by using the Scherrer's formula, and the narrower FWHM implicates the larger crystalline ordering [41]. It is therefore reasonable that the FWHM of a scattering peak of SAXS patterns can also be employed to evaluate the degree of ordering, especially the size distribution of periodic structures. Each SAXS scatting peak was fitted with a Lorentz function and the FWHM of each scattering peak along the radial direction in the scattering image was calculated from 2D microbeam SAXS patterns. Figure 3*a* presents the FWHM of pure HDPE and its nanocomposites at different distances away from the edge. It can be seen that the decrease of FWHM with the increase of distance is quite weak or can even be ignored for pure HDPE and the nanocomposites with lowest RGO content of 0.1 wt%, and this FWHW depression is just slightly accentuated with the increase of RGO content. More importantly, by comparing the results of the same distance of different nanocomposite bars, it is quite evident that the incorporation of RGO can depress the FWHW at each position, and the declining effect is remarkably accentuated with increasing RGO content. This can be seen more clearly in figure 3*b*, which shows the FWHM at the same distance of pure HDPE and its nanocomposites to further express the improvement of the orientation degree at different points with various RGO contents. These results indicate that RGO imposes no significant difference on the orientation ordering of polymer chains in the same sample at different positions. Nevertheless, the incorporation of RGO has remarkable effect for improving the orientation ordering of different injection bars.

**Figure 4.**
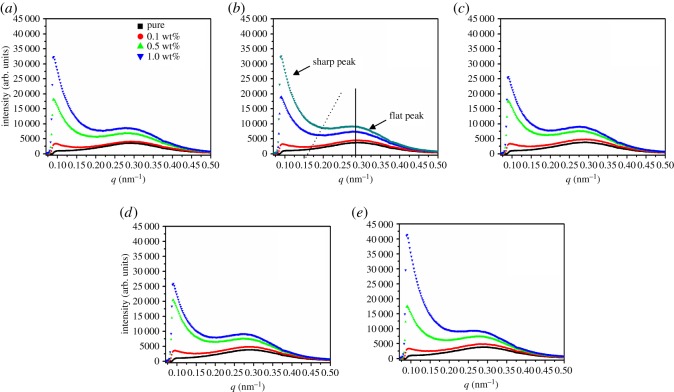
Radially integrated profiles of pure HDPE and its nanocomposites with different RGO contents at the location of the different distance to the sample edge: (*a*) 400 µm, (*b*) 800 µm, (*c*) 1200 µm, (*d*) 1600 µm and (*e*) 2000 µm.

To investigate the periodic structure of HDPE matrix, SAXS intensity profiles of pure HDPE and its nanocomposites are further presented. Figure 4 exhibits the radially integrated profiles of the meridian direction located at a different distance from the sample edge. To be consistent with the above discussion, we again select the RGO content of 1.0 wt% as the sample to analyse. With the increase of the distance to the edge, it is hard to find any change in the scattering intensity and peak position. These results are in agreement with above discussion (figures 1 and 2). Next, we again choose the location of 800 µm to discuss, as shown in figure 4*b*. The lamellae parameter derived from the SAXS data is the long period that can be calculated from the outer scattering ring (i.e. the flat peak in figure 4*b*) according to the Bragg equation. All flat peaks (solid line) show the same q value, indicating that all samples have the same long period and suggesting the formation of regular aligned crystal lamellae in HDPE matrix. Moreover, the sharp peak (imaginary line in figure 4*b*) that links up the central circle of 2D SAXS patterns can also be observed in the lower *q* value, and the *q* value of intensity valley increases with the addition of RGO. This result is in accordance with the figure 1, and has been discussed previously [36]. The phenomenon can be attributed to the dual role of epitaxial crystallization and space limitation during the cooling process in the presence of RGO [36]. Moreover, not only 800 µm but also other distances exhibit the same result, although we will not discuss this here. Based on the results, it is reasonable that HDPE chains can be absorbed upon the RGO surfaces and then grow into crystals in the overall positions of the sample.

**Figure 5.**
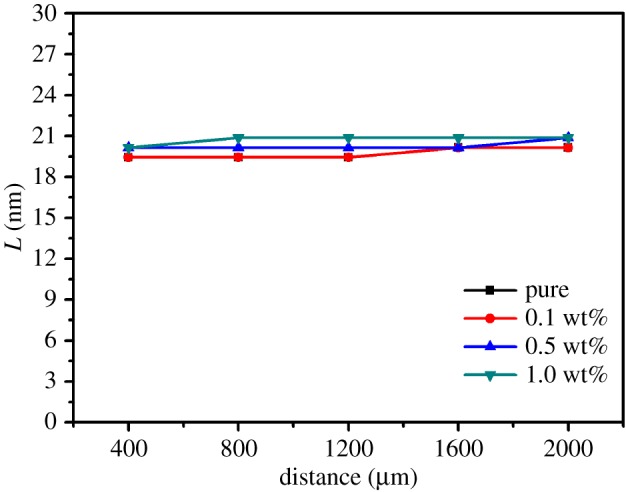
Long period profiles of pure HDPE and its nanocomposites with different RGO contents and with different regions.

To further distinguish the effects of RGO content on different distances, figure 5 quantitatively expresses the long periods of different regions with various RGO contents. From the outermost region to the innermost region, there is again no obvious change for the long period of each sample. Meanwhile, the long periods of different samples have only a slight difference at the same position, which can be ignored. These results are consistent with those of SAXS intensity profiles in figure 4. At the same distance to the outmost edge, the orientation of polymer chains is enhanced with the incorporation of RGO. The appearance of the periodic structure with the addition of RGO at the central circle of the 2D SAXS pattern is due to the fact that HDPE chains can be epitaxially crystallized upon RGO surfaces, and the enhancement of the periodic structure is owing to the increased space restriction with the increase of RGO contents [36].

It is now well accepted that the flow in the injection moulding process can induce the orientation of polymer chains along the flow direction, but the release of chain orientation also takes place at the same time [13–17]. Hence, the rapid cooling and therefore the fast crystallization in the surface layers can hold the preferred orientation caused by the shear flow, resulting in the formation of the skin and shear layers consisting of highly oriented lamellar crystals [13]. By contrast, the slow cooling and crystallization in the bulk and central parts provide enough time to allow the release of oriented polymer chains, leading to the generation of common spherulites [13]. It has been confirmed that the PE chains can be absorbed and then epitaxially crystallized on RGO surfaces. In the injection moulding process of HDPE/RGO nanocomposites, it is reasonable that the flow field can also drive the preferred orientation of both HDPE chains and RGO nanosheets [30]. However, in the subsequent cooling course, the situation becomes different from that of pure polymers, for the release of the oriented large RGO nanosheets is rather hard to achieve. Meanwhile, the HDPE chains are expected to be absorbed upon RGO surfaces during the melting and screwing process. Consequently, the epitaxial crystallization of absorbed HDPE chains on the oriented RGO surfaces results in the significant enhancement of the degree of preferred orientation of the injection bars of HDPE/RGO nanocomposite, and the intensifying effect is dramatically accentuated with the increase of RGO content, as has been discussed above. In addition, the slight enhancement of the orientation with the position close to the centre in HDPE/RGO nanocomposite bars can be attributed to the flow-caused slightly inhomogeneous distribution of RGO nanosheets. In the flow process of injection moulding, the centre has the faster flow rate, but the smaller shear force is expected to be more beneficial for the presence of large RGO nanosheets, while the opposite environment in the edge perhaps hinders the RGO location, which leads to a slightly inhomogeneous and enhanced distribution of RGO when approaching the centre and therefore the gentle enlargement of orientation with the increase of the distance from the edge, as illustrated in figure 3*a*. Moreover, this distribution is gently intensified with increasing RGO content, as illustrated in figure 3*b*. This point can also be confirmed by the approximately unvaried long period (as shown in figure 5), which indicates that the increase of oriented ordering cannot be attributed to the enlargement of crystal size, and thus the accumulation of preferred crystals should be responsible for the slight divergence of enhancing effect in different positions of the same HDPE/RGO bars. As has been noted previously, in the case of 0.5 wt% RGO, the FWHM in the distance of 400 is slightly smaller than that in the distance of 800 µm, indicating that the orientation of the edge is better. This effect can be explained as follows. At the edge, although the high shear force and the low flow rate hamper the location of RGO, they can enhance the orientation of RGO nanosheets more effectively. So, when the content of RGO is quite small, the less but highly oriented RGO can also lead to the gently preferred orientation. Recently, we also reported that the introduction of RGO can enhance the mechanical properties of the injection bars of HDPE/RGO nanocomposites [30]. Here, by further investigating the microstructures via microbeam SAXS, despite the presence of weak difference in the enhancing effect at varied positions, especially for the cases of large RGO contents, we have demonstrated that the coupling between the flow-induced orientation and the RGO-depressed release of oriented chains by absorption and then epitaxial crystallization imposes an approximately uniform intensifying effect on the crystallization and orientation of HDPE in the flow direction, which results in the enhancement of the mechanical performance of injection bars of HDPE/RGO nanocomposites.

## 4. Conclusion

We have successfully employed scanning microbeam X-ray scattering techniques to reveal the micrometer-scale inhomogeneous structural distribution of injection-moulded HDPE/RGO nanocomposite bars. SAXS results suggest that HDPE chains can be absorbed on the RGO surface and then grow into crystals via epitaxial crystallization in the cooling process, which induces the appearance of a novel periodic structure. Although flow field drastically affects the crystallization kinetics and crystal morphology of semicrystalline polymers during injection processing, the incorporation of RGO enhances the overall orientation degree of HDPE chains in injection bars but does not influence the crystal structure. In other words, for the same injection bar, the influence of the incorporation of RGO is not remarkable with the position modification. But for the different injection bars, the enhancement of crystallization and orientation is gradually increased at the equidistant position with the incorporation of RGO. This effect is applied to the whole sample and can also improve the mechanical properties of HDPE nanocomposites, which we have proved in our previous work. This research is expected to be helpful for understanding the enhancement mechanism of the layered nanofillers on the structures and corresponding mechanical properties of semicrystalline polymers. To enhance the understanding of the enhancement mechanism in polymer/nanofiller composites, especially for the nanofillers with space lattice matching to polymer, further studies with *in situ* observations of structural evolution in the stretching process of HDPE/RGO nanocomposite injection bars by online SAXS/WAXD measurements will provide us with useful information regarding the detailed mechanism of structural change upon elongation.

## Data accessibility

Data available from the Dryad Digital Repository: http://dx.doi.org/10.5061/dryad.p212867 [42].
